# Bdnf-Nrf-2 crosstalk and emotional behavior are disrupted in a sex-dependent fashion in adolescent mice exposed to maternal stress or maternal obesity

**DOI:** 10.1038/s41398-023-02701-1

**Published:** 2023-12-18

**Authors:** Chiara Musillo, Kerstin C. Creutzberg, Barbara Collacchi, Maria Antonietta Ajmone-Cat, Roberta De Simone, Marcello Lepre, Irmgard Amrein, Marco A. Riva, Alessandra Berry, Francesca Cirulli

**Affiliations:** 1 Center for Behavioral Sciences and Mental Health, Istituto Superiore di Sanità, Viale Regina Elena 299, 00161 Rome, Italy; 2 Ph.D. Program in Behavioral Neuroscience, Department of Psychology, Sapienza University of Rome, 00185 Rome, Italy; 3 Department of Pharmacological and Biomolecular Sciences, University of Milan, Milan, Italy; 4 National Center for Drug Research and Evaluation, Istituto Superiore di Sanità, Viale Regina Elena 299, 00161 Rome, Italy; 5 Functional Neuroanatomy, Institute of Anatomy, University of Zürich, Zurich, Switzerland; 6 Biological Psychiatry Unit, IRCCS Istituto Centro San Giovanni di Dio Fatebenefratelli, Brescia, Italy

**Keywords:** Molecular neuroscience, Predictive markers

## Abstract

Maternal obesity has been recognized as a stressor affecting the developing fetal brain, leading to long-term negative outcomes comparable to those resulting from maternal psychological stress, although the mechanisms have not been completely elucidated. In this study, we tested the hypothesis that adverse prenatal conditions as diverse as maternal stress and maternal obesity might affect emotional regulation and stress response in the offspring through common pathways, with a main focus on oxidative stress and neuroplasticity. We contrasted and compared adolescent male and female offspring in two mouse models of maternal psychophysical stress (restraint during pregnancy - PNS) and maternal obesity (high-fat diet before and during gestation - mHFD) by combining behavioral assays, evaluation of the hypothalamic–pituitary–adrenal (HPA) axis reactivity, immunohistochemistry and gene expression analysis of selected markers of neuronal function and neuroinflammation in the hippocampus, a key region involved in stress appraisal. Prenatal administration of the antioxidant N-acetyl-cysteine (NAC) was used as a strategy to protect fetal neurodevelopment from the negative effects of PNS and mHFD. Our findings show that these two stressors produce overlapping effects, reducing brain anti-oxidant defenses (*Nrf-2*) and leading to sex-dependent impairments of hippocampal *Bdnf* expression and alterations of the emotional behavior and HPA axis functionality. Prenatal NAC administration, by restoring the redox balance, was able to exert long-term protective effects on brain development, suggesting that the modulation of redox pathways might be an effective strategy to target common shared mechanisms between different adverse prenatal conditions.

## Introduction

Psychological stress or mental disorders affect about 10–15% of pregnant women worldwide, with important consequences for the developing offspring [1]. A variety of challenges, such as infection, air pollution, smoking, or dietary imbalance, have also been shown to disrupt the intrauterine environment, in turn, affecting fetal brain development [2–5]. In particular, metabolic stress during pregnancy, due to unhealthy nutritional habits, is emerging as a pressing public health issue.

Differently from the well-documented undernutrition suffered by pregnant women during World War II [6, 7], offspring development is nowadays threatened by prenatal exposure to “obesogenic environments” as a result of unlimited access to high-caloric “junk food” during pregnancy [5, 8]. A growing body of evidence is providing an association between maternal overnutrition or obesity and a higher risk for neurodevelopmental and psychiatric disorders in the offspring, including autism spectrum and attention-deficit hyperactivity disorders, cognitive deficits, and depression [5, 9, 10]. Interestingly, very similar effects also characterize offspring exposed to maternal psychological stress [11–13].

To explain how comparable negative consequences for offspring mental health can arise from prenatal stressors of different natures, we introduced a conceptual model known as the “funnel effect” [14]. This model hypothesizes that maternal psychological and metabolic stress converge, disrupting evolutionary-conserved stress-response pathways, negatively affecting fetal brain development, and increasing vulnerability to mental disorders. Numerous studies support an interdependent relationship among different stress–response systems, most notably involving redox regulations, inflammation, and neuroendocrine activation [15]. We have collected data to indicate that, in mouse models, both psychophysical stress and obesity independently lead to a pro-oxidant profile in maternal blood, with disrupted redox balance in the intrauterine milieu (Musillo et al. in preparation). Since reactive oxygen and nitrogen species are known to trigger inflammatory processes through the activation of NF-κB and the downstream cascades or the NLRP3 inflammasome [16, 17], this may expose the fetus to an increased inflammatory milieu. In addition, we have also shown that both maternal stress and maternal obesity independently exert similar detrimental effects by weakening the placental barrier, allowing excess levels of glucocorticoid hormones to reach the fetus, negatively affecting brain development [18, 19].

In this study, we specifically hypothesized that a disruption in the redox balance at the maternal-fetal interface could lead to a dysregulated amount of pro-inflammatory cytokines and glucocorticoids, overall affecting fetal brain development. We tested this hypothesis comparing the negative outcomes deriving from prenatal exposure to either psychophysical (maternal restraint) or metabolic (maternal high-fat diet - HFD) stressors in mouse offspring, assuming a central role for oxidative stress (OS) in funneling the effects of early adverse experiences on the developing brain [14, 20]. In the adolescent male and female offspring, we characterized emotionality and stress-activated pathways, including neuroendocrine as well as neuroplasticity and neuroinflammatory markers, all processes modulated by redox mechanisms. We focused on the hippocampal Bdnf-Nrf-2 crosstalk, which is an important effector of brain plasticity and is modulated by oxidative status [21]. To further dissect the role of OS in mediating the long-term effects of early life stressors, we tested whether maternal administration of N-acetyl-cysteine (NAC), a powerful anti-oxidant compound, might be used as a preventive strategy. Identifying a common pathway activated by different adverse conditions - often co-occurring within individuals - could lead to new early biomarkers for prevention screening and health promotion in populations at risk (mothers suffering from stress or obesity).

## Materials and methods

This study was reported in conformity with ARRIVE guidelines [22].

### Animal handling

One-month-old C57BL/6 N mice, 190 female and 95 male breeders, were purchased from Charles River (Italy) and sex-matched housed two/cage in transparent cages (Tecniplast), in an air-conditioned room (temperature 21 ± 1 °C, relative humidity 60 ± 10%), under a reversed 12/12 h light/dark cycle with lights off from 7 a.m. to 7 p.m. Fresh tap-water and standard diet (SD - energy 3.3 kcal/g, fat 17%, carbohydrate 60%, and protein 23%) were constantly available (Mucedola, Italy).

All experimental procedures were approved by the ethical body of the Istituto Superiore di Sanità for animal welfare, conducted in conformity with the European Directive 2010/63/EU and the Italian legislation on animal experimentation, D.Lgs. 26/2014 and authorized by the Italian Ministry of Health.

### Prenatal stressors

After three weeks of habituation, female breeders were allocated into the experimental groups based on a minimization approach, avoiding body weight bias [23].

#### Psychophysical stress (prenatal stress - PNS)

Pregnant females assigned to the PNS group (*n* = 20) were individually restrained in a transparent cylinder (11.5 × 3 cm) and contextually exposed to bright light as an additional stressor (6.500 lux) for 30 min, three times daily, from gestational day (G) 12.5 until G18.5. Stress sessions were conducted during the dark phase, at different times during the day to prevent habituation to the repeated procedure [24]. Control females (CTRL, *n* = 27) were left undisturbed during the pregnancy period.

#### Metabolic stress (maternal HFD - mHFD)

Female breeders were assigned to the control diet (CD, *n* = 47) or the high-fat diet (HFD, *n* = 48) groups. Diets were administered ad libitum for 10 weeks before mating and throughout gestation (for a total of 13 weeks) until G16, when both HFD and CD were replaced with SD to prevent cannibalistic behaviors and pups’ mortality [25]. HFD (D12331, energy 5.56 kcal/g, fat 58%, carbohydrate 25.5% and protein 16.4) and CD (D12328, energy 4.07 kcal/g, fat 10.5%, carbohydrate 73.1%, and protein 16.4%) were provided by Research Diets Inc., USA.

### NAC administration and breeding procedure

Five weeks before mating, female breeders were divided into two groups receiving either the antioxidant NAC (*n* = 95: 47 for the PNS model; 48 for the mHFD model) or tap water as a vehicle (*n* = 95: 47 for the PNS model; 48 for the mHFD model). NAC was administered daily in drinking water to yield an average dose of 1 g/kg body weight [20] until G16 for a total of 8 weeks. NAC was purchased as powder by Sigma Aldrich.

After 5 weeks of NAC administration (and 10 weeks on diets for the mHFD cohort), females were bred. A handful of dirty sawdust (urine) from a male’s cage was added to a female’s cage every morning for 3 days so that pheromones might induce the estrus cycle. On the third day, one male was introduced into each female’s cage for 48 h. Thereafter, the weight gain of females was monitored to confirm pregnancy and to determine the length of gestation with a precision of ±0.5 day. A weight gain of at least 3 g on G7 usually indicates that conception has occurred.

The final number of pregnant females within each experimental group was as follows: CTRL-Vehicle = 14; CTRL-NAC = 11; PNS-Vehicle = 11; PNS-NAC = 11; CD-Vehicle = 13; CD-NAC = 13; HFD-Vehicle = 12; HFD-NAC = 12.

### Experimental procedures on the offspring

**Fig. 1:**
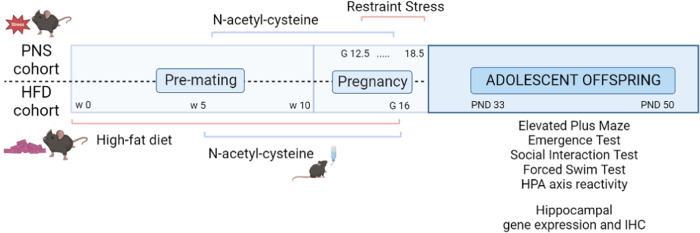
Experimental design. Abbreviations: PNS prenatal psychophysical stress, HFD high-fat diet, w week, G gestational day, PND post-natal day, HPA hypothalamic–pituitary–adrenal, IHC immunohistochemistry.

Pups’ birth was considered as post-natal day 0 (PND 0). One or two males and one or two females, when possible, were selected from each litter for the behavioral characterization. Litters with pups ≤3 were excluded. At PND 10 male and female offspring were tested in the Homing test, at PND30 weaned and from PND 33 until PND 50 tested to assess the emotional profile, the sociability, and the neuroendocrine reactivity in response to an acute stress. The experimental design of this study is shown in Fig. 1.

### Behavioral phenotype

All tests were conducted during the dark phase and video-recorded. The behavioral analysis was performed by an observer blind to the experimental conditions using a dedicated video tracking software: The Observer XT 15, Noldus (the Netherlands), and AnyMaze (Stoelting, USA). All apparatuses were cleaned with a cotton pad and 70% ethanol solution between each session.

#### Homing test

On PND 10, when the eyes were still closed, pups were tested in the Homing test. This test was used to assess mouse general neurodevelopment since it requires the proper integration of sensory-motor, associative, and discriminative capabilities [26], and it is based on the tendency of pups to maintain contact with the mother. The latency to reach the nest zone was measured (see Supplementary Fig. 1 for details).

#### Elevated plus maze

This test was used to evaluate the emotional profile, exploration, and risk-assessment behaviors. The apparatus was made of Plexiglas (dim grey floor, transparent walls) and raised to a height of 60 cm above the floor level, with two open (30 × 5 cm) and two enclosed arms (30 × 5 × 15 cm) extending from a common central platform (5 × 5 cm). Mice were individually placed on the central platform facing an open arm and were allowed to freely explore for 5 minutes (100 lux). The time spent in the open vs. the closed arms was assessed [27]. The frequency of rearing, wall-rearing, and head dipping - three main exploratory behaviors [28] - were used to calculate a composite index of exploratory activity by means of *Z*-score, while the frequency of stretched attend posture (SAP) was analyzed as a measurement of risk assessment.

#### Emergence test

The apparatus was a cubic arena (40 × 40 × 40 cm), ideally divided into 25 squares partitioned into a central zone (24 × 24 cm) and a peripheral zone (the remaining part of the arena). A shelter (black plastic cup - 15 cm diameter) in one corner provided a retreat possibility from the brightly lit arena (600 lux) [29]. Mice started the test inside the shelter and the latency to emerge was evaluated as an index of emotionality. Mice were free to explore for 20 min: distance traveled, mean speed, and the time spent in the different zones were measured.

#### Social interaction test

To increase social behaviors, mice were individually housed 24 h before the test. During the test, mice were placed in a novel cage with an unfamiliar conspecific, sex- and weight-matched (tail marked by a nontoxic paint) for a total of 20 min. We calculated a composite *Z*-score for social behaviors (frequency of behaviors directed to the conspecific: snout sniffing, body sniffing, and anogenital sniffing). The duration of rearing and wall-rearing was analyzed as an index of vertical exploration.

#### Forced swim test and coping stress strategy

This test was used to evaluate the coping strategies in response to an acute stress. Mice were gently placed into a cylindrical transparent tank (30 cm h x 20 cm diameter) filled with water (26 ± 1 °C) up to 25 cm, so mice were not able to touch the bottom of the tank. One session of 6 min was performed, and only the last 4 min were analyzed [30]. The % time spent performing the following strategies was assessed: passive strategy (floating); and active strategy (swimming and struggling).

### Plasma corticosterone levels in response to forced swim test

Activation of the hypothalamic–pituitary–adrenal (HPA) axis was assessed in response to an inescapable stressful challenge. Blood samples were collected by a tail nick before the stress (baseline) as well as 30 and 180 min following the stress exposure. Blood samples were collected in potassium EDTA-coated tubes, and plasma was separated by centrifugation at 3000 rpm for 15 min at +4 °C and immediately stored at −80 °C. Corticosterone (CORT) levels were determined using commercial ELISA kits (Enzo Life Sciences, USA).

### Brain tissue collection

At PND 50, male and female offspring were sacrificed by cervical dislocation, brains removed from the skull, and hippocampi dissected from the left hemispheres and immediately frozen at −80 °C until gene expression analysis. The right hemispheres were post-fixed in 4% paraformaldehyde (PFA) overnight at +4 °C and then stored in a 0.05 Sodium Azide solution until immunohistochemical analysis.

### RNA extraction and gene expression analysis

Total RNA was isolated from left hippocampi using the RNeasy Mini Kit (Qiagen) according to the manufacturer’s protocol. RNA concentration was measured with a NanoDrop spectrophotometer (Thermo Fisher) and used for quantitative real-time polymerase chain reaction (qRT-PCR) (CFX384 real-time system, Bio-Rad Laboratories). Samples were run in triplicate and β-actin was used as a housekeeping gene. Primers for Nuclear factor erythroid 2-related factor 2 (Nrf-2); Kelch Like ECH Associated Protein 1 (Keap-1); cluster of differentiation 68 (Cd 68), triggering receptor expressed on myeloid cells 2 (Trem 2), transmembrane protein 119 (Tmem 119), inducible nitric oxide synthase (iNOS), arginase 1 (Arg-1), mitochondrial uncoupling protein 2 (Ucp- 2), tumor necrosis factor-α (Tnf-α), interleukin 6 (Il-6), transforming growth factor beta (Tgf-β), insulin-like growth factor 1 (Igf-1) were purchased from Thermo Fisher Scientific while for β-actin and Bdnf total, primers and probes were purchased from Eurofins Genomics. Primer sequences and ID assay are listed in Supplementary Table 1. All analyses were performed following the delta-delta CT method with β-actin as endogenous control. Data are presented as fold change % compared to the control group (set at 100%).

### Immunohistochemistry

Post-fixed right hemispheres were coded to conduct the immunohistochemistry (IHC) procedure and analysis. Brains were placed in 20% glycerol solution at 4 °C overnight for cryoprotection and then embedded in gelatin-egg-albumin blocks (4 brains in each block) [31]. Each block was cut into 40 µm-thick coronal sections using a freezing microtome (HYRAX S 30); ten complete series were collected in cryoprotection solution and stored at −20 °C until use. One complete series was mounted in the correct anatomical order (reference series), and Giemsa was stained. Sections containing the hippocampus were used for IHC Iba-1 (ionized calcium-binding adapter molecule 1) staining. For IHC staining, one complete series of free-floating sections was first rinsed in Tris-Triton and incubated in citrate buffer [0.1 M] at 95 °C for 40 min to retrieve antigens. After cooling down, the sections were treated with 0.6% peroxidase solution for 15 min and rinsed again in Tris-Triton. They were then placed in a blocking buffer of 2% normal goat serum (NGS) and 0.2% Triton in Tris-Triton for 1 h. Afterward, sections were incubated with anti-IBA1 antibody (anti-rabbit, 1:3000, Wako) at 4 °C overnight. The next day, sections were rinsed in Tris-buffered saline (TBS) and incubated with a goat-anti-rabbit secondary antibody (1:300, Reactolab) for 40 min; rinsed again in TBS and incubated with ABC solution (Reactolab) for 20 min. After additional rinses, sections were stained with DAB (Sigma-Aldrich) for 4 min, mounted, Giemsa counterstained, and embedded.

### Quantitative analysis of Iba1-positive cells

We focused on the CA1 and the dentate gyrus (DG) of the hippocampus, two main sub-regions that have been associated with microglial alterations in animal models of early life stress [32, 33]. Quantification of Iba1-positive cells in these regions was performed by an observer, blind to the experimental conditions, using the optical fractionator probe [34, 35], with a section sampling fraction of 10, step size of 250 μm, and frame size of 90 × 90 μm. On average, 6 sections per animal contained the CA1 and the DG regions. Iba1-positive cells were identified as ramified (multiple long processes) or intermediate (multiple short processes). Iba1-positive cell number estimates were performed with StereoInvestigator 10 software (MBF Bioscience, Williston, VT, USA) on a Zeiss microscope (Zeiss, Germany) using a 40× oil immersion lens. Sections were analyzed in the correct anatomical order, and cell numbers from each section were then standardized into 2 virtual sections representing the dorsal (rostral) and ventral (intermediate/temporal) CA1 region [36]. Analysis was done blind to animal identity in 6–7 animals per sex and experimental group.

### Statistical analysis

Statistical analysis was performed using GraphPad Prism version 9 (GraphPad Software, USA). The sample size was calculated through the G*Power 3.1 software (www.gpower.hhu.de). Bartlett’s test was used to test the homogeneity of variances among groups. When a main effect of sex was found, male and female mice were separately analyzed by means of a two-way ANOVA with prenatal condition (PNS/CTRL or HFD/CD) and prenatal treatment (NAC/Vehicle) as between-subject factors and repeated measures as within-subject factors (i.e., time, zones of the apparatus, cell morphology). Post hoc comparisons among groups were performed using Tukey’s test, also in the absence of significant ANOVA effects according to the indications given by Wilcox [37]. For those outcomes that did not follow a normal distribution (CORT), data were normalized by means of the square root of the raw values. The cumulative incidence of latency was analyzed using a log-rank (Mantel–Cox) test [38] with the application of Bonferroni’s correction (Homing and Emergence tests). Grubb’s test, using 5% significance level critical values, was used to detect outliers [39]. A level of probability set a *p* < 0.05 was used as statistically significant.

## Results

### Early assessment of neurodevelopment through the Homing test at PND10

Neurodevelopment assessed during the early postnatal phases indicated that PNS females showed the most vulnerable phenotype.

#### PNS cohort

PNS improved the ability to reach the nest zone, specifically in females, by reducing their latency. Interestingly, prenatal NAC prevented this effect (see Supplementary Fig. 2 for details).

#### mHFD cohort

No significant effects were found upon mHFD or prenatal NAC in both sexes.

### Characterization of the emotional profile during adolescence

Strong sex-dependent effects characterized both PNS and mHFD female offspring, supporting the “funnel effect” model.

### Emergence test

#### PNS cohort

**Fig. 2:**
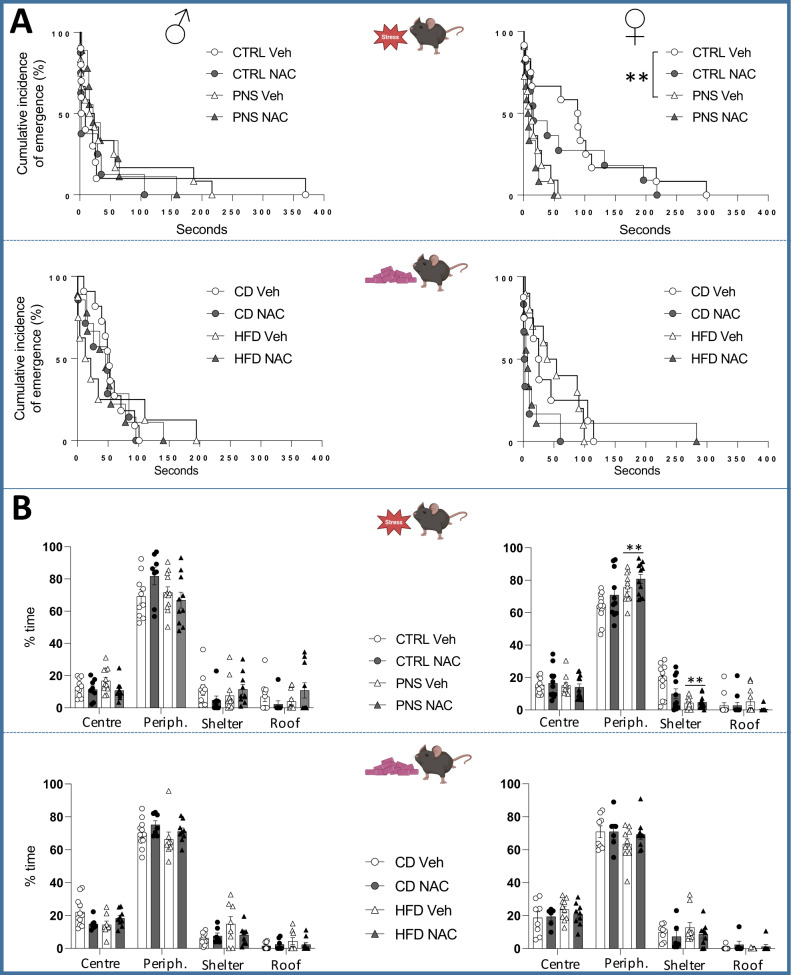
Emotional behavior in the emergence test. **A** PNS reduced the latency to emerge from the shelter, specifically in female offspring, while no effects were observed upon mHFD. Each dot represents the cumulative incidence of emerging from the shelter over the course of the test; ##*p* < 0.01 log-rank (Mantel–Cox) test with Bonferroni correction. **B** PNS reduced total time spent in the shelter and increased time in the periphery, while no effects were observed in mHFD. Data are mean ± SEM; **p < 0.01 Tukey’s test PNS vs. CTRL. Number of subjects: 6–12 within each experimental group. C center, P periphery, S shelter, R roof of the shelter.

PNS affected female offspring only, reducing the latency to emerge from the shelter compared to the CTRL group (*χ*^2^ = 8.364, *p* = 0.0038, Fig. 2A). Sex-dependent effects were also found when the time spent in the different zones of the apparatus was evaluated: PNS females reduced the time spent inside the shelter but increased the time in the periphery of the arena compared to CTRL subjects (stress × zones: *F*(3,126) = 9.585, *p* < 0.0001; post hoc comparisons: *p* < 0.01 PNS vs. CTRL), see Fig. 2B. Furthermore, PNS females increased speed and distance traveled (mean speed: *F*(1,42) = 5.675, *p* = 0.0218; distance: *F*(1,42) = 5.485, *p* = 0.0240; Supplementary Table 2). The maternal administration of NAC did not affect the parameters assessed.

#### mHFD cohort

No difference between groups was observed when assessing the latency to emerge from the shelter. A preference for the peripheral zone was observed for all subjects (males: *F*(3,93) = 519.339, *p* < 0.0001; females: *F*(3,87) = 374.592, *p* < 0.0001, see Fig. 2A and Supplementary Table 2). Overall, neither the mHFD nor the prenatal NAC affected the behaviors assessed in the emergence test in both sexes.

### Elevated plus maze - EPM

#### PNS cohort

**Fig. 3:**
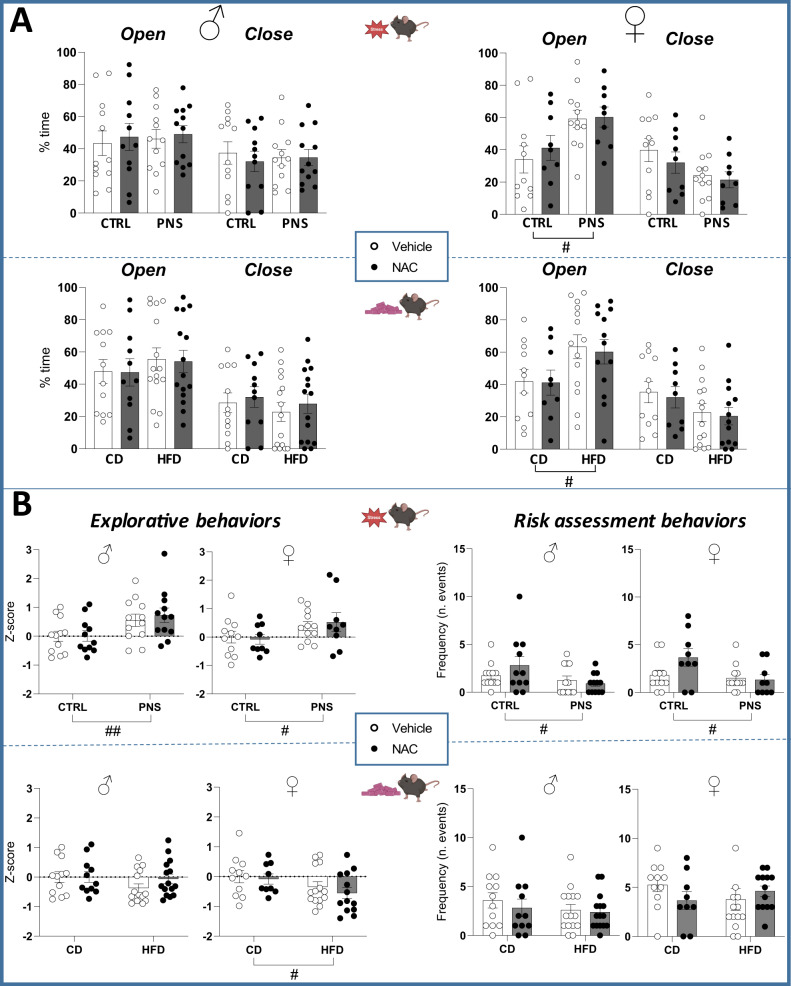
Emotional and explorative behaviors in the EPM. **A** Prenatal exposure to either PNS or mHFD resulted in similar effects on the emotional profile of female offspring, increasing the time spent in the open arms. **B** Overall, PNS increased exploration and reduced risk assessment behaviors, while mHFD reduced exploratory behaviors only in females and did not affect risk assessment. #*p* < 0.05, ##*p* < 0.01 main effect of prenatal stress/diet. Data are mean ± SEM. Number of subjects: 9–15 within each experimental group.

All adolescent mice showed a preference for the open arms (females: *F*(1,37) = 9.401, *p* = 0.0040; males: *F*(1,43) = 3.612, *p* = 0.0641), a behavior often observed during adolescence. Moreover, PNS females spent more time in the open arms (stress × arm: *F*(1,37) = 7.867, *p* = 0.0080; post hoc comparisons: *p* < 0.05 PNS vs. CTRL; Fig. 3A). Exploratory activity was increased by PNS in both sexes (females: *F*(1,37) = 5.136, *p* = 0.0294; males: *F*(1,43) = 9.290, *p* = 0.0039), while the frequency of risk assessment behaviors was reduced (females: *F*(1,37) = 4.947, *p* = 0.0323; males: *F*(1,43) = 5.015, *p* = 0.0304; Fig. 3B). In this test, the prenatal NAC did not affect the parameters assessed.

#### mHFD cohort

Also in this cohort, all adolescent mice preferred open arms (females: *F*(1,43) = 12.891, *p* = 0.0008; males: *F*(1,49) = 12.848, *p* = 0.0008). Mirroring the PNS, mHFD increased the time spent in the open arms, specifically in females (diet × arm: *F*(1,43) = 5.827, *p* = 0.0201; post hoc comparisons: *p* < 0.05 HFD vs. CD; Fig. 3A). However, mHFD decreased exploratory activity in female mice (*F*(1,43) = 4.611, *p* = 0.0374), while no changes were observed in risk assessment behaviors (Fig. 3B). In this test, the prenatal NAC did not affect the parameters assessed.

### Characterization of sociability during adolescence in the Social interaction test

#### PNS cohort

PNS decreased social behaviors in female subjects, while prenatal NAC administration increased exploratory behaviors both in females and males (see Supplementary Fig. 3 for details).

#### mHFD cohort

Prenatal NAC was able to boost social behaviors in males exposed to mHFD (see Supplementary Fig. 3).

### Behavioral strategy and neuroendocrine reactivity in response to stress

Adolescent male offspring exposed to PNS or mHFD showed reduced basal CORT levels, in line with the “funnel effect” model. The modulation of the redox balance through NAC administration was able to normalize these effects.

### Coping stress strategy in the forced swim test

#### PNS cohort

**Fig. 4:**
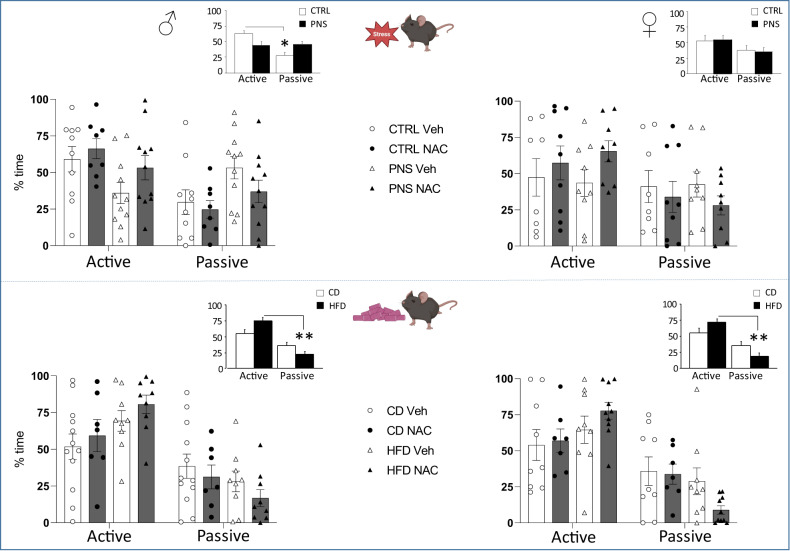
Coping strategies in response to acute stress. PNS males failed to show a clear preference for a coping strategy towards stress when compared to the CTRL group. mHFD exacerbated the preference for adopting an active coping strategy towards stress in both sexes (see the Inset representing the interaction between stress and strategy). *p < 0.05 Tukey’s test active CTRL-Veh vs. passive CTRL-Veh; **p < 0.01 males active HFD-Veh vs passive HFD-Veh; females active HFD-Veh vs. passive HFD-Veh. Data are mean ± SEM. Number of subjects: 7–11 within each experimental group.

Dealing with an acute inescapable stress, CTRL male offspring showed a clear preference towards an active coping strategy (stress × strategy: *F*(1,36) = 5.341, *p* = 0.0267; post hoc comparisons: *p < 0.05 CTRL-Veh active vs. passive). By contrast, PNS males spent an equal amount of time displaying active as well as passive strategies (Fig. 4). No significant differences were observed in female offspring exposed to different prenatal conditions.

#### mHFD cohort

Exposure to mHFD increased the time spent performing an active coping strategy at the expense of a passive strategy in both sexes (diet × strategy - males: *F*(1,33) = 3.901, *p* = 0.0567; females: *F*(1,31) = 3.997, *p* = 0.0544; post hoc comparisons: **p < 0.01 HFD-Veh active vs. passive, see Fig. 4). No significant changes were observed as a result of prenatal NAC administration.

### HPA axis reactivity

#### PNS cohort

**Fig. 5:**
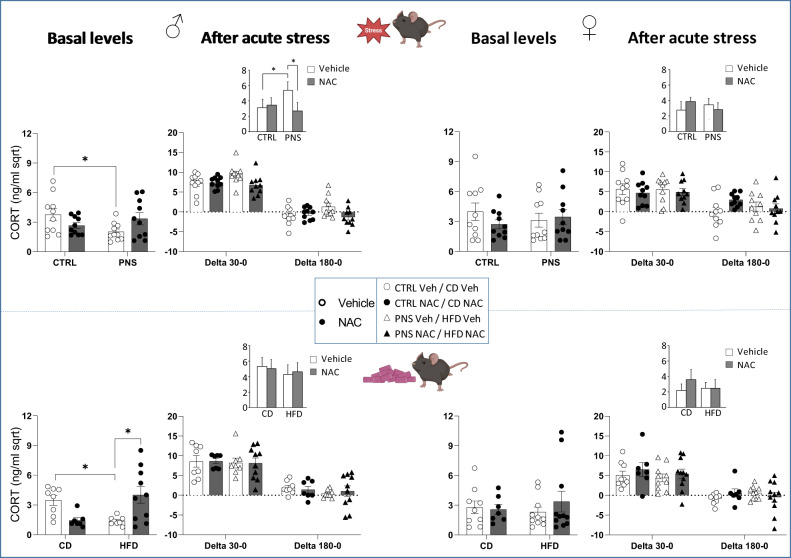
HPA axis under basal conditions and its reactivity in response to acute stress. PNS resulted in lower basal CORT levels in male offspring. When the reactivity of the HPA axis was evaluated 30 and 180 min after the exposure to acute stress, PNS males were characterized by overall higher CORT levels and prenatal NAC was effective in buffering CORT rise (see the Inset representing the interaction between stress and treatment). HFD reduced basal CORT levels in male offspring. Prenatal NAC administration was able to prevent this effect. *p < 0.05 Tukey’s test males PNS-Vehicle vs. CTRL-Vehicle; PNS-NAC vs. PNS-Vehicle; HFD-Vehicle vs. CD-Vehicle; HFD-NAC vs. HFD-Vehicle. Data are mean ± SEM. Number of subjects: 7–11 within each experimental group.

When CORT levels were assessed under basal conditions, we found that PNS exposure reduced basal CORT levels in male offspring only (stress × treatment: *F*(1,36) = 6.353, *p* = 0.0163; post hoc comparisons: *p < 0.05 PNS-Veh vs. CTRL-Veh), see Fig. 5. Next, the reactivity of the HPA axis was evaluated 30 and 180 min following an acute stress (FST). Overall, PNS males reacted to acute stress with enhanced CORT release after 30 minutes and they still showed higher circulating levels after 180 min from the stress, when CORT is expected to return to a baseline (stress × treatment: *F*(1,36) = 5.585, *p* = 0.0236; post hoc comparisons: *p < 0.05 PNS-Veh vs. CTRL-Veh, see the inset in Fig. 5). Interestingly, prenatal NAC buffered CORT rise in PNS males (*p < 0.05 PNS-NAC vs. PNS-Veh, Fig. 5). We did not observe differences in CORT levels of female offspring.

#### mHFD cohort

Mirroring the PNS cohort, exposure to mHFD led to reduced CORT levels under basal conditions in male mice (diet × treatment: *F*(1,29) = 15.49, *p* = 0.0005; post hoc comparisons: *p < 0.05 HFD-Veh vs. CD-Veh, Fig. 5). Prenatal NAC was able to restore basal CORT levels (*p < 0.05 PNS-NAC vs. PNS-Veh, Fig. 5). As for HPA axis reactivity after acute stress, no changes were observed in both male and female offspring (Fig. 5).

### Neuroprotective and neuroinflammatory gene expression in the hippocampus of adolescent offspring

To identify molecular changes underlying PNS and mHFD, we investigated the hippocampal gene expression of some prototype genes that indicate the functional status of different systems, including the neurotrophins *Igf-1* and *Bdnf* for neuronal plasticity; the transcription factor *Nrf-2* and its chaperone *Keap-1* for redox balance; the microglia/macrophage markers *Cd 68*, *Tmem 119* and *Trem 2*; the neuroinflammatory markers *iNOS*, *Arg-1, Ucp-2*, and the inflammatory cytokines *Tnf-α*, *Il-6*, and *Tgf-β*.

The most interesting result, supporting the “funnel effect” of different early stressors, is the mirroring decrease of *Bdnf* and *Nrf-2* levels found in the adolescent female offspring exposed to PNS or mHFD. Moreover, both PNS and mHFD lastingly affected key homeostatic functions of hippocampal microglia. The restorative effects of NAC administration corroborate the main role played by the redox balance in mediating these early stressors.

#### PNS cohort

**Fig. 6:**
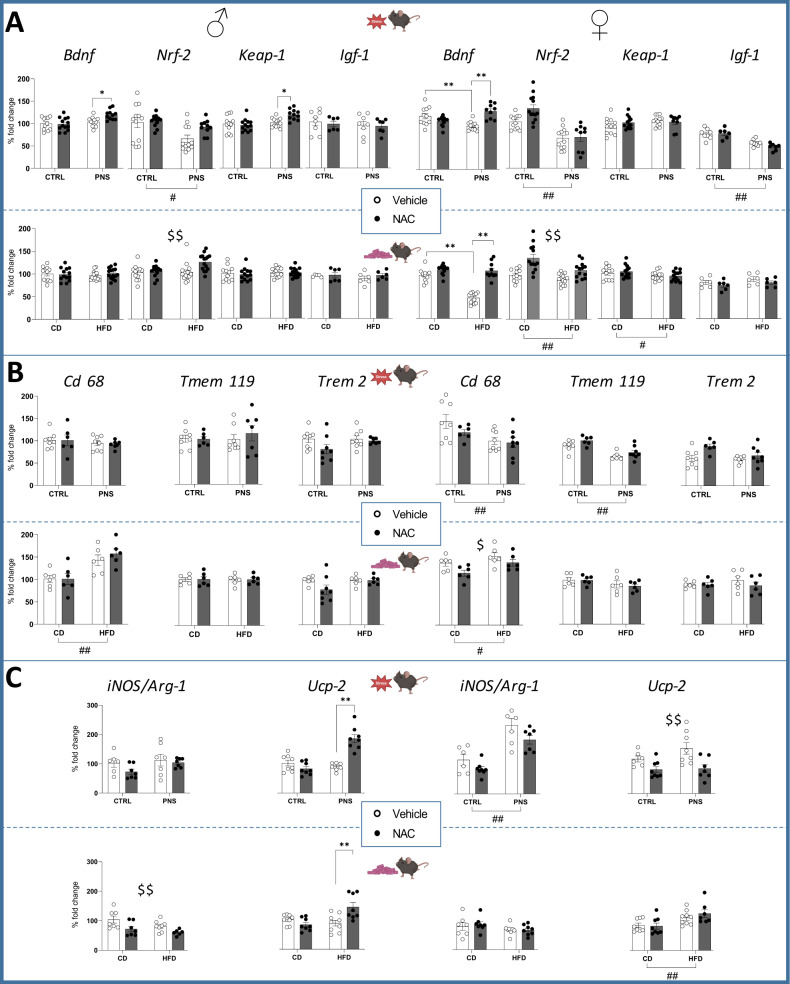
Hippocampal expression of genes involved in plasticity, redox mechanisms, microglia, and neuroinflammatory-related markers. **A** Both PNS and HFD resulted in a similar reduction of hippocampal *Bdnf* and *Nrf-2*, with greater effects on females. Overall, PNS also reduced *Igf-1* expression in female offspring. **B** While PNS reduced *Cd 68* and *Tmem 119* levels specifically in females, mHFD overall increased *Cd 68* both in males and females. **C** PNS increased *iNOS/Arg1* in female offspring. Prenatal NAC overall reduced Ucp-2 expression. In females, mHFD upregulated *Ucp-2*. In males, expression of *Ucp-2* was increased in PNS-NAC as well as in HFD-NAC groups specifically. #*p* < 0.05, ##*p* < 0.01 main effect of prenatal stress/diet; $*p* < 0.05, $$*p* < 0.01 main effect of NAC; *p < 0.05, **p < 0.01 Tukey’s test. Data are mean ± SEM. Number of subjects: 6–12 within each experimental group.

A decrease in total *Bdnf* mRNA levels in PNS females was found (stress × treatment *F*(1,40) = 24.36, *p* < 0.0001); post hoc comparisons: **p < 0.01 PNS-Veh vs. CTRL-Veh). Interestingly, prenatal NAC was able to prevent this effect, restoring *Bdnf* levels (**p < 0.01 PNS-NAC vs. PNS-Veh, Fig. 6A). The evaluation of *Igf-1* levels revealed an overall decrease in PNS females (stress: *F*(1,25) = 46.79, *p* < 0.0001; Fig. 6A) that was not prevented by NAC administration. As for the redox regulations, PNS greatly decreased hippocampal *Nrf-2* expression, regardless of sex (females: *F*(1,42) = 46.05, *p* < 0.0001; males: *F*(1,43) = 10.10, *p* = 0.0027, Fig. 6A). No difference was observed in *Keap-1* mRNA levels in female offspring. In general, less pronounced effects were observed in male offspring. Prenatal NAC increased total *Bdnf* levels in PNS subjects only (stress × treatment *F*(1,43) = 5.240, *p* = 0.027; post hoc comparisons: *p < 0.05 PNS-NAC vs. PNS-Veh). When evaluating the expression levels of *Keap-*1, an increase in PNS-NAC group compared to PNS-Vehicle was found in male offspring (stress × treatment: *F*(1,44) = 6.450, *p* = 0.0147; post hoc comparisons: *p < 0.05 PNS-NAC vs. PNS-Veh, Fig. 6A).

When assessing macrophages/microglia-related markers, again, we found sex-dependent effects, which were magnified in female offspring. Hippocampal levels of *Cd 68* and *Tmem 119* RNAs were decreased by PNS, specifically in females (*Cd 68*: *F*(1,26) = 8.349, *p* = 0.0077; *Tmem 119*: *F*(1,24) = 31.46, *p* < 0.0001, Fig. 6B) with no effect of prenatal NAC.

As for the neuroinflammatory mediators, we found that PNS overall increased *iNOS/Arg-1* ratio in females (stress: *F* (1,25) = 34.44, *p* < 0.0001) by increasing *iNOS* and reducing *Arg-1* expression (*iNOS:* *F*(1,25) = 40.97, *p* < 0.0001; *Arg-1:* *F*(1,25) = 8.211, *p* = 0.0083); the prenatal NAC increased *Arg-1* (*F*(1, 25) = 7.472, *p* = 0.0113) although the *iNOS/Arg-1* ratio was not significantly reduced. PNS did not alter *Ucp-2* expression in females, while prenatal NAC overall reduced it (*F*(1,25) = 12.76, *p* = 0.0015, Fig. 6C). No changes of *iNOS/Arg-1* expression were observed in male offspring, while *Ucp-2* was upregulated in the PNS-NAC group (stress × treatment: *F*(1,27) = 33.75, *p* < 0.0001, post hoc comparisons: **p < 0.01 PNS-NAC vs. PNS-Veh, Fig. 6C).

In addition, the analysis of the cytokines *Tnf-α*, *Il-6*, *Tgf-β* revealed no significant PNS- or NAC-induced changes in either sex, except for an overall increase of *Tgf-β* in NAC-treated females (see Supplementary Fig. 4).

#### mHFD cohort

As in the PNS cohort, we found decreased total *Bdnf* levels as a result of mHFD, specifically in females (diet × treatment (*F*(1,41) = 34.69, *p* < 0.0001; post hoc comparisons: **p < 0.01 HFD-Veh vs. CTRL-Veh), an effect prevented by prenatal NAC administration (**p < 0.01 HFD-NAC vs. HFD-Veh, Fig. 6A). In parallel, mHFD female offspring were characterized by lower levels of *Nrf-2* (*F*(1,47) = 10.99, *p* = 0.0018), NAC treatment, also in this case, preventing these effects (*F*(1,47) = 27.04, *p* < 0.0001, Fig. 6A). *Keap-1* levels were reduced specifically in mHFD female subjects (*F*(1,45) = 4.673, *p* = 0.0360). As for male offspring, while no changes were found in total *Bdnf, Igf-1*, or *Keap-1*, a general increase of *Nrf-2* mRNA levels was observed upon prenatal exposure to NAC (*F*(1,51) = 7.715, *p* = 0.0076, Fig. 6A).

Concerning macrophage/microglial specific markers we found an increase in *Cd 68* levels both in males and females exposed to mHFD (males: *F*(1,20) = 20.27, *p* = 0.0002); females: *F*(1,20) = 6.699, *p* = 0.0176), see Fig. 6B. Prenatal NAC was able to prevent this effects only in females (*F*(1,20) = 6.004, *p* = 0.0236). No significant changes were observed in mRNA levels of *Tmem 119* or *Trem 2*.

Furthermore, mHFD, while upregulating *Arg-1* expression, did not decrease the *iNOS/Arg-1* ratio in females (*F*(1,27) = 10.33, *p* = 0.0034). mHFD also upregulated *Ucp-2* expression in females (*F*(1,28) = 11.96, *p* = 0.0018; see Fig. 6C). In male offspring, prenatal NAC reduced *iNOS* expression (F(1,24) = 7.522, p = 0.0113) as well as *iNOS/Arg-1* ratio (*F*(1,24) = 9.709, *p* = 0.0047). Similarly to the PNS cohort, an increase of *Ucp-2* expression was found in the HFD-NAC male group (stress × treatment: *F*(1,29) = 12.47, *p* = 0.0014, post hoc comparisons: **p < 0.01 HFD-NAC vs HFD-Veh, Fig. 6C).

In addition, a reduction of *Il-6* by both mHFD and NAC was found in females only (Supplementary Fig. 4).

### Microglia morphology and distribution in the hippocampal CA1 and DG

We estimated the number and the morphology of Iba1-positive cells in the CA1 and DG of the hippocampus, two main sub-regions that have been associated with microglial alterations in animal models of early life stress [32, 33]. We took into account the dorsoventral distribution, as the dorsal and the ventral portions of the hippocampus are mainly involved in different functions: the first, named “*the cold,*” plays a role in regulating cognitive function, while the second one, “*the hot*” is involved in modulating emotional processes [40].

#### PNS cohort

There were no differences in the total number of microglial cells in the CA1 or DG between groups. In males, the exposure to PNS combined with NAC treatment reduced the number of ramified microglia cells (“surveilling” phenotype) in the ventral CA1, compared to prenatal NAC alone (*F*(1,21) = 4.680, *p* = 0.0422; *p < 0.05 Tukey’s test PNS-NAC vs. CTRL-NAC, Supplementary Fig. 5).

#### mHFD cohort

Similarly, in the mHFD cohort, no changes were found in the total number of microglial cells in CA1 or DG. In males, the mHFD combined with NAC treatment reduced the number of ramified “surveilling” microglia in the dorsal CA1 compared to prenatal NAC alone (**p < 0.01 Tukey’s test HFD-NAC vs. CD-NAC, see Supplementary Fig. 5).

## Discussion

In the present study, we provide evidence supporting the hypothesis that both psychophysical and metabolic stress independently act through a “funnel effect” model, triggering shared pathways and redirecting fetal developmental trajectories, leading to increased vulnerability to negative behavioral outcomes in a sex-dependent fashion [14]. NAC administration was able to prevent some of these effects, corroborating the notion that the modulation of OS-related pathways effectively targets a mechanism shared between different adverse prenatal conditions.

The first evidence supporting a “funnel effect” model is a parallel reduction in hippocampal *Bdnf* mRNA levels in adolescent females as a result of two different prenatal conditions, namely PNS and mHFD. This sex-dependent effect indicates that, at least during adolescence, female mice are more vulnerable to prenatal stressors, confirming and expanding our previous findings [41, 42]. It is of interest that a reduction in hippocampal levels of *Nrf-2*, a master regulator of antioxidant defenses, mirrored *Bdnf* changes. Surprisingly, expression of *Igf-1* did not change, except for PNS females, indicating that the Bdnf-Nrf2 crosstalk and, in turn, redox balance, is the main common mechanism underlying PNS and mHFD, independently from other metabolic factors.

The emotional behavior of the adolescent offspring also showed strong, sex-specific differences, with larger effects in female mice, overall characterized by behavioral disinhibition. In particular, in both PNS and mHFD, females increased the time spent in the open arms of the EPM, as well as exploration and decreased risk-assessment behaviors. This profile was also observed in the Emergence and in the Homing tests in PNS females. Overall, behavioral “disinhibition”, which includes a broad spectrum of behavioral traits, such as impulsiveness, low levels of control, and increased novelty seeking, may be considered an adaptive strategy to meet immediate emotional demands in a novel unfamiliar context, although it might set the stage for later adverse functional outcomes and psychopathology [43–45].

The association of increased behavioral disinhibition with low hippocampal *Bdnf* levels in female offspring exposed to PNS or mHFD is in line with the “stress acceleration hypothesis” [46]. According to this idea, early adversities might prematurely activate neuronal structures involved in emotional regulation and stress response, such as the hippocampus [46]. In an evolutionary framework, an overall accelerated development of specific circuits would confer an advantageous emotional adaptation and behavioral flexibility characterized by a rapid response to new conditions and less anxiety in the short term, also allowing a higher reproductive success and fitness in adverse conditions [47–49]. A downregulation of *Bdnf-Nrf2* is, however, suggestive of reduced neuroplasticity that may hold long-term negative consequences on emotional behavior as *Bdnf-Nrf2* mutual crosstalk has been indicated as involved in the etiopathogenesis of mood disorders [21, 50, 51].

Stress during pregnancy has been shown to alter HPA axis activity in the offspring, although findings appear heterogeneous. While some preclinical and clinical studies have reported that early life stress leads to a hyper-activation of the HPA axis, other evidence indicates lower basal cortisol levels or a blunted cortisol response to acute stress [52–55]. One possible explanation is based on the concept that disruptions of offspring HPA axis due to maternal stress can depend upon the developmental stage, as shown by studies from the ALSPAC cohort reporting higher cortisol awakening response in 10-years-old children but lower levels of the same parameter at age 15 [56, 57].

According to previous evidence, our data indicate reduced basal CORT levels in adolescent male offspring exposed to PNS or mHFD, NAC administration preventing this reduction. This effect, which corroborates the “funnel effect” model, was specifically found in male subjects, strengthening the notion that PNS and mHFD converge on common mechanisms in a sex-dimorphic manner. Exposure to PNS also led to an impairment in coping strategies and a more prolonged elevation of CORT levels in response to a stressful challenge, which may be suggestive of impaired negative feedback, an effect largely normalized by NAC administration.

As for the potential mechanisms, our group has previously shown that both PNS and mHFD are able to weaken the placental barrier by reducing the activity of 11β-Hydroxysteroid dehydrogenase 2 (11β-HSD 2), allowing an excessive amount of glucocorticoids to reach the fetus, potentially affecting HPA axis development [19, 25]. A similar reduction in 11β-HSD2 placental levels has been observed as a result of prenatal exposure to OS, an effect counteracted by NAC administration through inhibition of OS-induced PERK/p-eIF2α signaling cascade in the placenta of male fetuses [58–60]. Thus, activation of this cascade might represent a specific common mechanism underlying male-specific HPA axis dysfunction.

Within the fetal brain, microglia are the main sensor of environmental factors that can affect their ability to regulate developmental neurogenesis and brain plasticity [61]. Since newborn microglial cells can be easily primed by an altered intrauterine environment [32, 62], we assessed whether long-lasting alterations of the hippocampal microglial immune profile (as assessed by analysis of *Tmem 119, Trem 2, and Cd 68* expression), and OS/neuroinflammatory status (as assessed by *Il-6*, *Tnf-α*, *Tgf-β*, *iNOS*, *Arg-1*, *and Ucp-2* transcript analysis) could be detected at the critical developmental window of adolescence. We specifically selected these genes for their increasingly recognized involvement in the microglial-mediated modulation of proper developmental brain trajectories.

Results indicate that both PNS and mHFD lastingly affect key homeostatic functions of hippocampal microglia of adolescent offspring, in line with previous studies in similar rodent models [33, 63, 64].

Interestingly, the two prenatal stressors promoted distinct neuroinflammatory profiles, suggesting different microglial priming programs, with most of the molecular alterations affecting female offspring. Specifically, in PNS females, we observed a decrease of *Cd 68*, a marker of immune/phagocytic activation also involved in synaptic pruning, accompanied by reduced levels of the microglial surface receptor *Tmem 119*, a putative marker of microglial homeostatic function involved in the regulation of cell survival [65, 66]. A higher ratio of *iNOS/Arg-1*, commonly taken as an index of microglia pro-inflammatory polarization, was also found despite unchanged levels of *Tnf-α*, *Il-6*, and *Tgf-β*.

A different scenario was elicited by mHFD, which increased hippocampal *Cd 68* levels in both male and female adolescent offspring. In addition, in females only, mHFD decreased *Il-6* expression and induced *Arg-1* as well as *Ucp-2*, a negative regulator of reactive oxygen species, which plays a key role in microglia activation, mitochondrial dynamics, and function [67]. Likewise, prenatal NAC modulated hippocampal neuroinflammatory gene expression in a sexually dimorphic manner. In line with its anti-oxidant and anti-inflammatory activity, NAC overall increased *Arg-1* and *Tgf-β* and reduced *Ucp-2* expression in the female PNS cohort, while it decreased *Cd 68* and *Il-6* expression in females and reduced the *iNOS/Arg-1* ratio in males, in the mHFD cohort. The NAC-dependent upregulation of *Nrf-2* observed in both PNS and HFD offspring may be responsible for these changes, as Nrf-2 modulates the inflammatory response by several mechanisms, including the crosstalk with the NF-kB and the inflammasome pathways [68].

As for the morphological characterization of Iba1+ cells, while no difference emerged in the DG, subtle changes were observed in the hippocampal CA1 subregion. In detail, NAC treatment, in combination with both PNS and mHFD, reduced the surveilling cell population in male offspring. Interestingly, this parallel effect was specifically detected in the ventral subregion (emotional domain) in PNS, while mHFD was found in the dorsal subregion (cognitive domain), leading to hypothesize that, while PNS might predispose to a major vulnerability for emotionality, mHFD might preferentially affect cognitive functions later in life.

Overall, in this study, we identified two clear sex-dimorphic phenotypic pathways affected by both PNS and mHFD: disrupted neuroendocrine regulations characterize long-term effects in males, while inflammatory-redox balance and emotionality are impaired in females. The preventive effect shown by NAC on many of the outcomes tested in this study supports the main role of OS as a converging mechanism between metabolic and psychological stressors. These promising findings might lead to the development of early prevention strategies specifically targeting stress or obesity during pregnancy. They also provide mechanistic insight into the comorbidity often reported between psychiatric and metabolic disorders.

### Supplementary information

Supplementary material

## Data Availability

All data needed to evaluate the conclusions in the paper are present in the paper and/or the Supplementary Materials. Additional data are available from authors upon request.
