# Supplementary material for: Bdnf-Nrf-2 crosstalk and emotional behavior are disrupted in a sex-dependent fashion in adolescent mice exposed to maternal stress or maternal obesity
Source: Transl Psychiatry. 2023 Dec 18;13:399. doi: 10.1038/s41398-023-02701-1 (PMC10725882; doi:10.1038/s41398-023-02701-1)
Supplement: Supplementary file 1 — Supplementary material [file 41398_2023_2701_MOESM1_ESM.docx]

**Supplementary Material**

**Supplementary Figures**

**
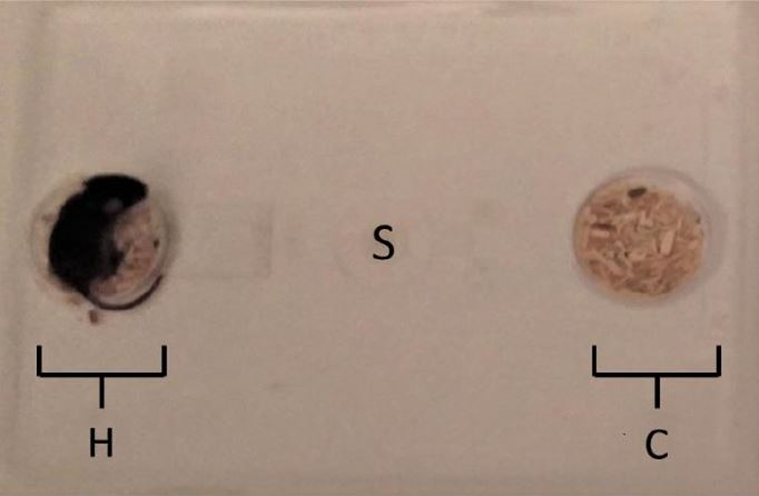
**

**Supplementary Figure 1 Experimental setting of the Homing test.** One male and one female from each litter were separated from the dam for 20 minutes and were placed in two holding cages at controlled temperature of 30±1°C. Thereafter, each pup was individually placed at the center of the arena and the latency to reach the nest zone was measured. The arena (20 x 15 cm) contained two equally-distanced-mini petri dishes (35 mm diameter) filled with 0.6 g of home cage material (nest zone) or clean sawdust (control zone), placed on opposite sides (Supplementary Figure 1). Abbreviations: H=nest zone filled with home cage material; C=control zone filled with clean sawdust; S=starting point.

*
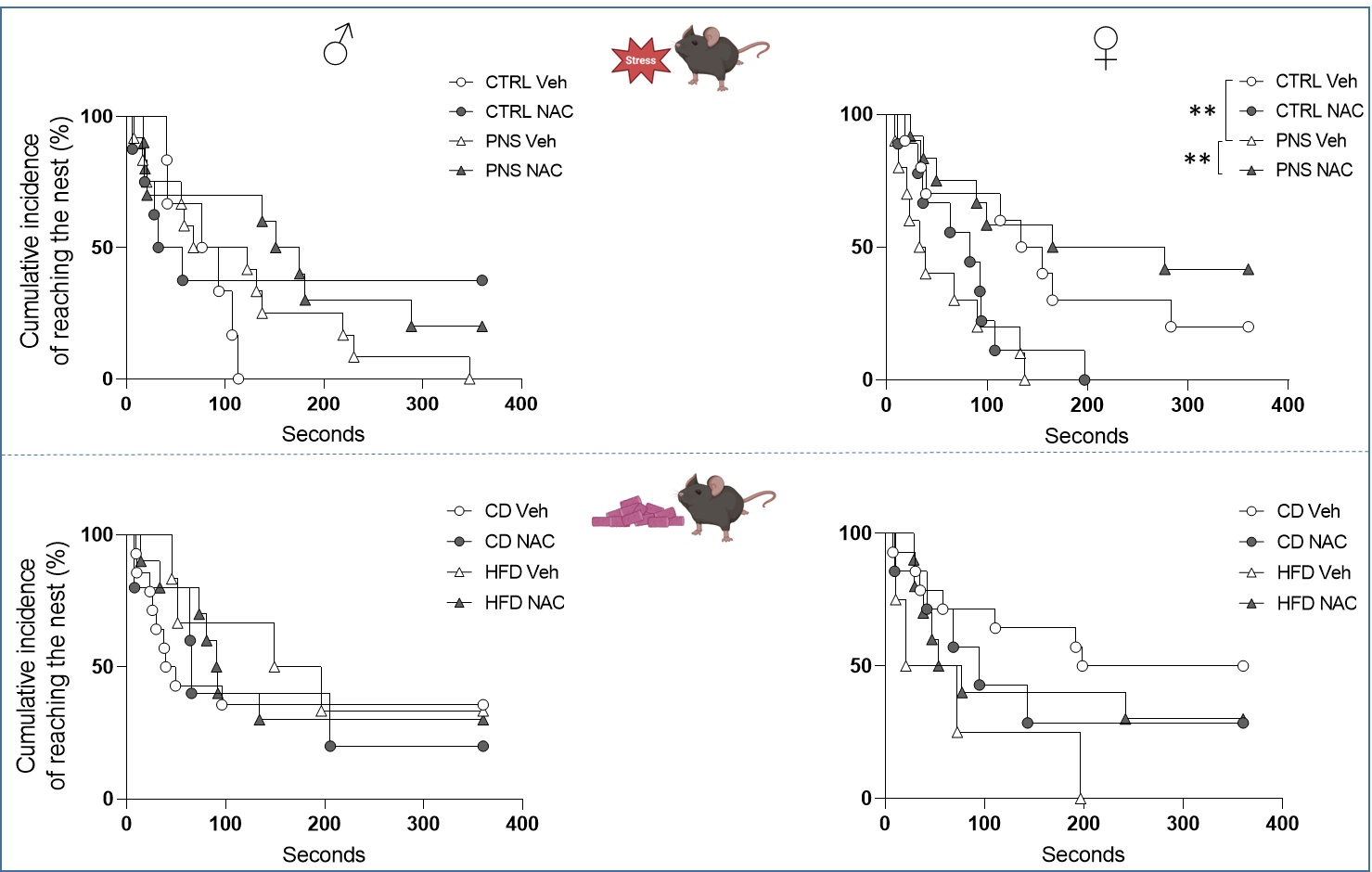
*

**Supplementary Figure 2 Early assessment of neurodevelopment through the Homing test.** PNS reduced the latency to reach the nest zone specifically in female pups (χ2=15.33, p=0.0016; Bonferroni’s correction: χ2=6.988, p=0.0082 PNS-Veh vs CTRL-Veh), an effect prevented by prenatal NAC administration (Bonferroni’s correction: χ2=9.248, p=0.0024). Each dot represents the cumulative incidence of emerging from the shelter over the course of the test; **p<0.01 log-rank (Mantel-Cox) test with Bonferroni correction; Number of subjects: 4-14 within each experimental group.


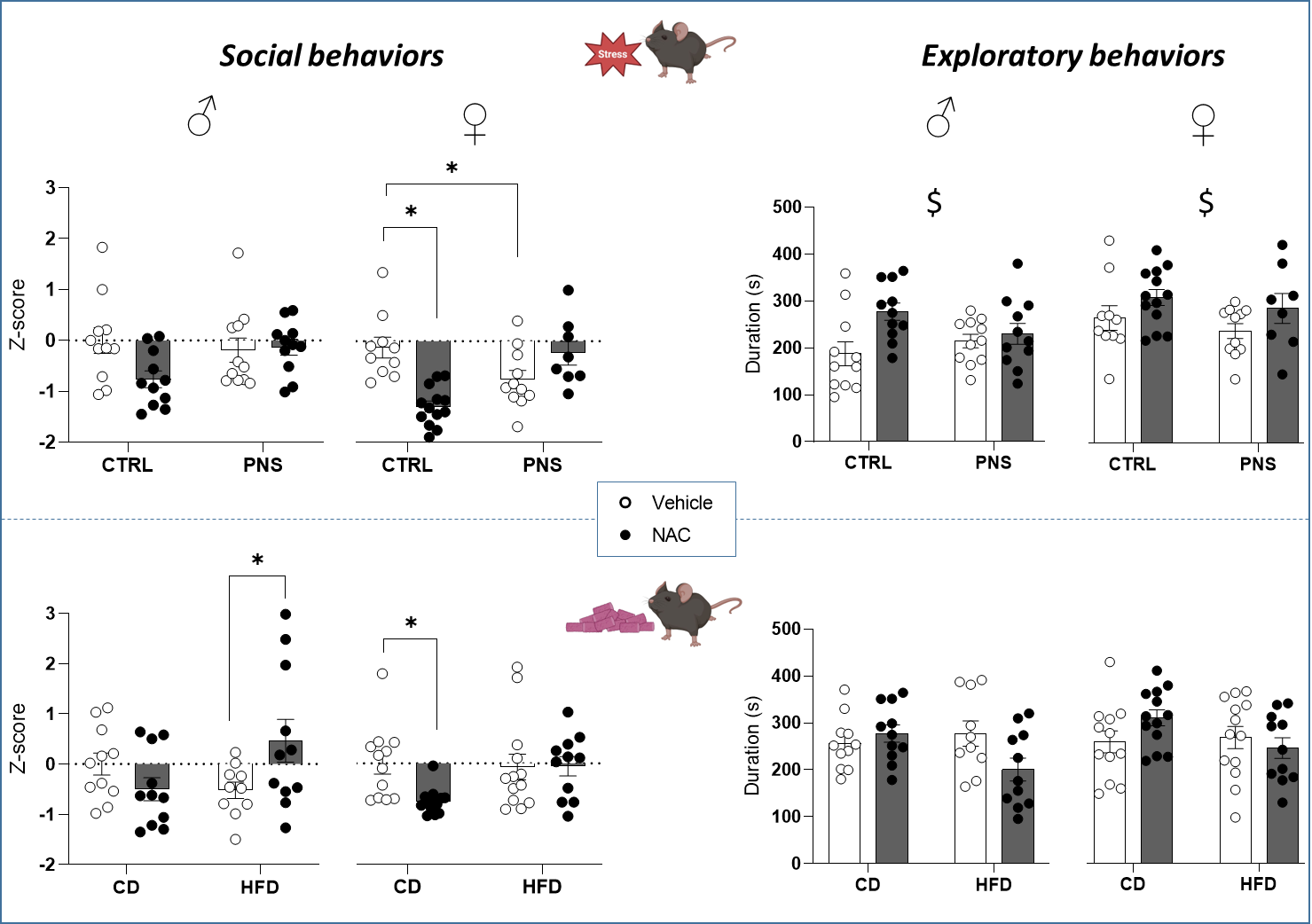


**Supplementary Figure 3 Social behaviors.** PNS decreased social behaviors specifically in female offspring (stress x treatment: F(1,38)=22.67, p<0.0001; post hoc comparisons: *p<0.05 Tukey’s test PNS-Veh vs CTRL-Veh). Prenatal NAC decreased social behaviors in females (*p<0.05 CTRL-NAC vs CTRL-Veh) in favor of increased exploratory behaviors (F(1,38)=4.216, p=0.0470). Prenatal NAC increased exploration also in males (F(1,40)=6.499, p=0.0147). In the mHFD cohort, prenatal NAC boosted social behaviors in HFD-NAC males (diet x treatment: F(1,39)=6.962, p=0.0119; *p<0.05 Tukey’s test HFD-NAC vs HFD-Veh), while decreasing these in CD-NAC females (F(1,45)=3.904, p=0.0543; *p<0.05 Tukey’s test CD-NAC vs CD-Veh).

**
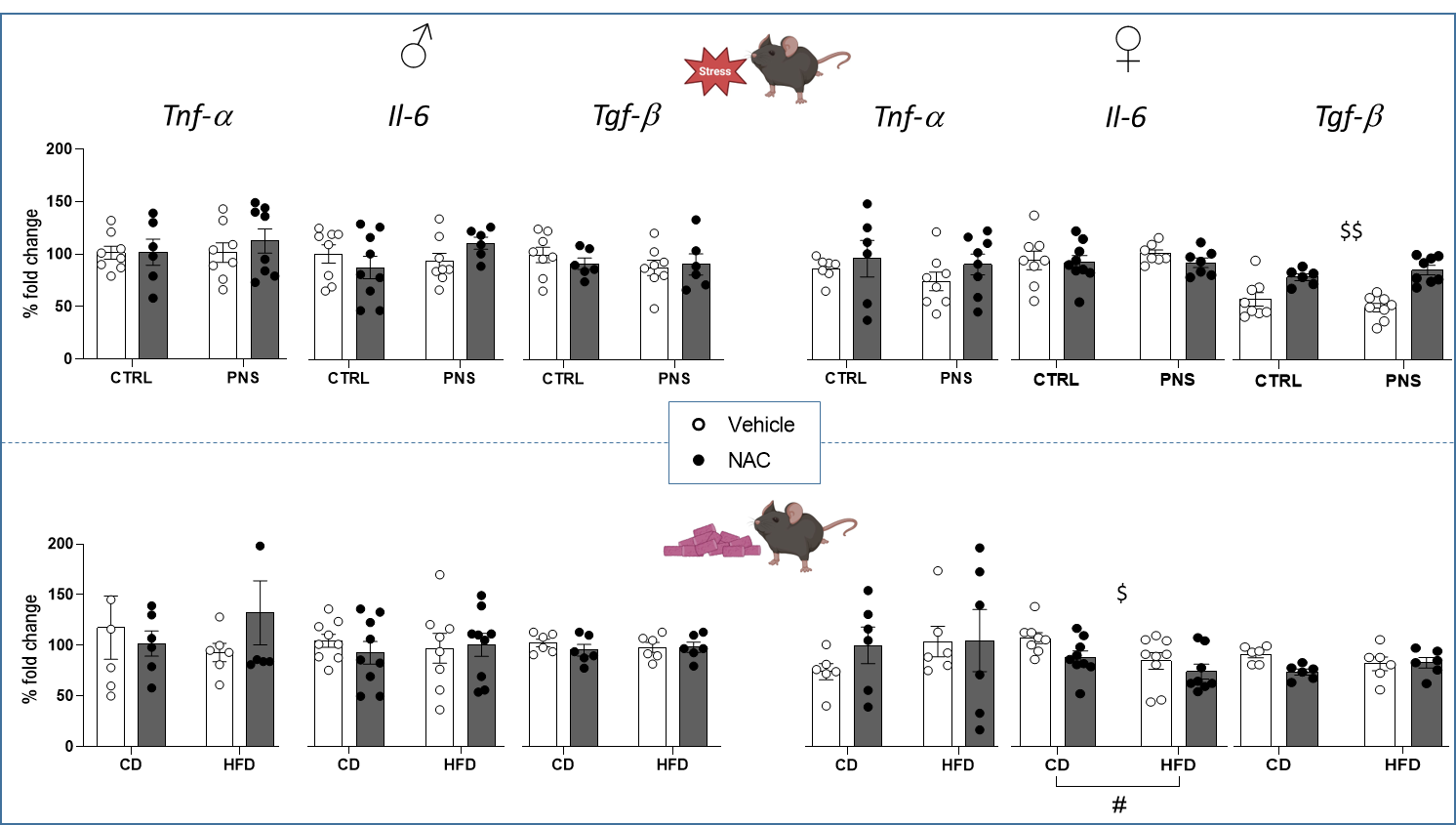
**

**Supplementary Figure 4 Neuroinflammatory markers.** PNS cohort: a main effect of treatment was found showing that NAC increased *Tgf-β* in females (F(1,26)=33.89, p<0.0001). mHFD cohort: both mHFD and NAC reduced *Il-6* levels in females (F(1,30=6.959, p=0.0131 and F(1,30)=4.47, p=0.0429, respectively). #p<0.05 main effect of diet; $p<0.05, $$p<0.01 main effect of NAC. Data are mean ± SEM. Number of subjects: 6-9 within each experimental group.


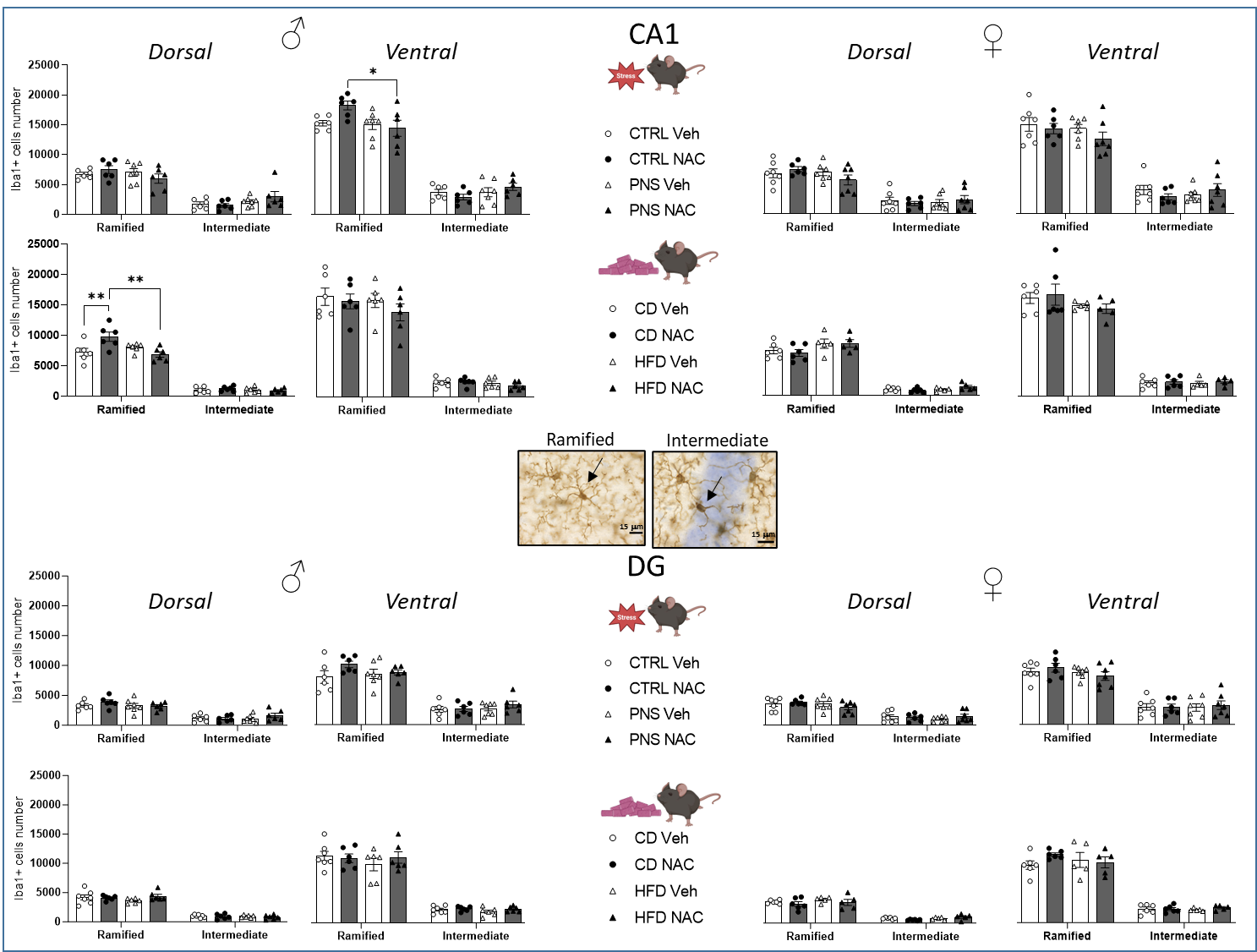


**Supplementary Figure 5 Stereological counting and morphological analysis of Iba1-positive cells in the CA1 and DG.** Exposure to PNS or mHFD, combined with NAC treatment, reduced the number of ramified microglia cells compared to prenatal exposure to NAC alone. In addition, CD-NAC males had a higher number of ramified microglial cells than CD-Veh males (**p<0.01 Tukey’s test CD-NAC vs CD-Veh). *p<0.05, **p<0.01 Tukey’s test. Data are mean ± SEM. Number of subjects: 5-6 within each experimental group.

**Supplementary Tables**

**Supplementary Table 1** Primer sequences and ID assay used for qRT-PCR analysis

| **Gene** | **Primer sequence and ID assay** |
| --- | --- |
| β-actin | Fwd: ACCTTCTACAATGAGCTGCG  Rev: CTGGATGGCTACGTACATGG  Probe: TCTGGGTCATCTTTTCACGGTTGGC |
| Bdnf total | Fwd: AAGTCTGCATTACATTCCTCGA  Rev: GTTTTCTGAAAGAGGGACAGTTTAT  Probe: TGTGGTTTGTTGCCGTTGCCAAG |
| Nrf-2 | Mm00477784_m1 |
| Keap-1 | Mm00497268_m1 |
| Igf-1 | Mm00439560_m1 |
| Cd68 | Mm03047343_m1 |
| Tmem 119 | Mm00525305_m1 |
| Trem 2 | Mm04209424_g1 |
| Arg-1 | Mm00475988_m1 |
| iNOS | Mm00440502_m1 |
| Ucp2 | Mm00627599_m1 |
| Tgf-β | Mm01178820_m1 |
| Il-6 | Mm00446190_m1 |
| Tnf-α | Mm00443258_m1 |

**Supplementary Table 2** Locomotor activity in the Emergence test

|  | **Distance (m)** | | **Speed (cm/s)** | |
| --- | --- | --- | --- | --- |
|  | **Males** | **Females** | **Males** | **Females** |
| CTRL-Vehicle | 32.43 ± 2.84 | 32.06 ± 1.48 | 2.69 ± 0.24 | 2.67 ± 0.13 |
| CTRL-NAC | 30.77 ± 2.51 | 34.33 ± 2.66 | 2.56 ± 0.21 | 2.85 ± 0.22 |
| PNS-Vehicle | 33.09 ± 1.94 | 37.45 ± 2.40 | 2.75 ± 0.16 | 3.12 ± 0.20 |
| PNS-NAC | 29.76 ± 2.54 | 41.73 ± 3.79 | 2.49 ± 0.21 | 3.48 ± 0.31 |
| CD-Vehicle | 38.20 ± 1.35 | 35.63 ± 1.47 | 3.18 ± 0.11 | 2.96 ± 0.13 |
| CD-NAC | 36.41 ± 2.92 | 39.76 ± 2.70 | 3.03 ± 0.24 | 3.30 ± 0.22 |
| HFD-Vehicle | 33.67 ± 2.33 | 37.14 ± 2.48 | 2.82 ± 0.19 | 3.09 ± 0.21 |
| HFD-NAC | 39.86 ± 2.34 | 41.90 ± 4.06 | 3.31 ± 0.19 | 3.50 ± 0.33 |

Data are mean ± SEM. Number of subjects: 6-12 within each experimental group
